# A systematic revision of the genus *Neopterygosoma* Fajfer, 2019 (Acariformes: Pterygosomatidae) with the description of a new species

**DOI:** 10.1007/s11230-020-09938-0

**Published:** 2020-09-30

**Authors:** Monika Fajfer

**Affiliations:** Department of Molecular Biology, Genetics and Immunology, Institute of Biological Sciences, Cardinal Stefan Wyszynski University, Wóycickiego 1/3, 01–938 Warsaw, Poland

## Abstract

A systematic revision of the scale mites of the genus *Neopterygosoma* Fajfer, 2019 (Acariformes: Pterygosomatidae) formerly placed in the genus *Pterygosoma* Peters, 1849, is presented. Two new natural species groups are established: the *chilensis* group for species found on Chilean liolaemid lizards (Sauria: Liolaemidae) and the *patagonica* group for *N*. *patagonica* (Dittmar de la Cruz, Morando & Avila, 2004) found on several *Liolaemus* spp. from Argentina. A neotype of *N*. *patagonica* is designated. A leg chaetotaxy model for tarsi-coxae I–IV is proposed for the genus. A key to all species of *Neopterygosoma* is provided and a full list of *Neopterygosoma* spp. with their updated host associations and distribution data is compiled. Additionally, a new species, *N. schroederi* n. sp. found on *Liolaemus schroederi* Müller & Hellmich, is described, including for the first time, description and illustrations of the immature stages of a species of *Neopterygosoma*.

## Introduction

The family Pterygosomatidae is represented by highly specific ectoparasites of lizards (Sauria) distributed throughout the world, except for Antarctica. Mites of the genus *Pimeliaphilus* Trägårdh, 1905 are only found on terrestrial arthropods (Paredes-León et al., 2012) and *Geckobia enigmatica* Bertrand & Pedrono, 1999 is found on tortoises (Testudines: Testudinae) (Bertrand & Pedrono, 1999).

Mites of the genus *Neopterygosoma* Fajfer, 2019 are associated with lizards of the genus *Liolaemus* (Sauria: Liolaemidae), and until recently they were placed in the genus *Pterygosoma* Peters, 1849 (see Fajfer & González-Acuña, 2013). In 2019, Fajfer reconstructed the phylogeny of the genus *Pterygosoma* based on the external morphology of the species using modern phylogeny methods (i.e. maximum parsimony and implied weighting) (Fajfer, 2019). As a result, the new genus *Neopterygosoma* was created for mites associated with South American lizards (Liolaemidae: *Liolaemus*). Currently, the genus includes six monoxenous species found on Chilean liolaemids, and one oligoxenous species, *N. patagonica* Dittmar de la Cruz, Morando & Avila, 2004, observed on several *Liolaemus* spp. (see Fajfer & González-Acuña, 2013). Until now, all the species descriptions were based solely on females, whereas immatures and males were not found.

This paper describes *Neopterygosoma schroederi* n. sp. found on *Liolaemus schroederi* Müller & Hellmich (Sauria: Liolaemidae). The deutonymph, protonymph and larva are described for the first time for a species of the genus *Neopterygosoma*. The diagnosis for the genus is provided in Fajfer (2019), therefore it is not repeated here. However, two natural species groups are established for the genus and the updated diagnoses of all species are proposed. Additionally, a neotype is designated for *N. patagonica*. The leg chaetotaxy model for the genus is presented and a key to the species of *Neopterygosoma* is constructed. A full list of host-parasite associations is compiled.

## Materials and methods

*Collection of material*

**Fig. 1:**
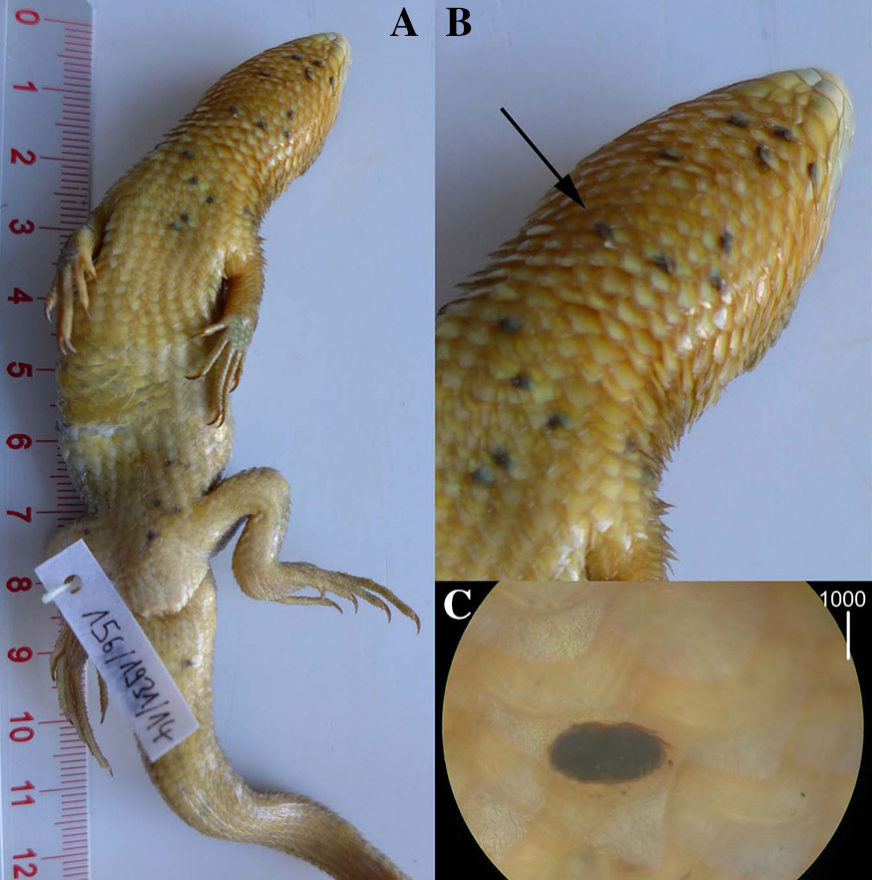
*Liolaemus chilensis* (Lesson) bearing pterygosomatid mites *Neopterygosoma chilensis* (Fajfer & González-Acuña, 2013). A, Ventral view; B, Ventral view of the head and neck; *arrow* indicates mites under the scales; C, A single specimen under the host scale. *Scale-bar*: C, 1000 µm

The type-material of *Neopterygosoma* spp. was loaned from the AMU (Adam Mickiewicz University in Poznań, Poznań, Poland), ZISP (Zoological Institute of the Russian Academy of Sciences, St. Petersburg, Russia) and ZMUC (Zoological Museum, University of Copenhagen, Copenhagen, Denmark). The new material used in this study was taken from dead lizards kept in jars with 70% ethanol in the ZSM (Bavarian State Collection of Zoology, Munich, Germany) and the NHM (Natural History Museum, London, UK). Each lizard’s body was completely and carefully checked for mites under a dissecting microscope (Fig. 1).

*Technique of slide mounting*

All mites were collected from the lizard specimens kept in the institutions mentioned above and most of the loaned type-specimens were preserved in 70% ethanol. Then, the mites were cleared and softened in Nesbitt’s solution at 45°C for 1–2 h and mounted in Hoyer’s medium on a glass slide using the standard method (Krantz & Walter, 2009). The prepared slides were dried with the thermostat set at 50–55°C for 7–10 days, and studied using the microscope Olympus CKX41 with the Olympus cellSens Standard 1.16 software.

*Terminology*

The names of the leg and idiosomal setae in species descriptions follow Grandjean (1939, 1944) as described by Norton (1977), while the names of the palpal setae follow Grandjean (1946). Grandjean’s nomenclature was applied to the family Pterygosomatidae by Bochkov & OConnor (2006).

The scientific names of the lizards follow Uetz et al. (2019).

**Family Pterygosomatidae Oudemans, 1910**

**Genus** ***Neopterygosoma*** **Fajfer, 2019**

**Species group** ***chilensis***

Diagnosis

Body much wider (1.5–1.8 times) than long. Second pair of legs discernible shorter than others. Postero-medial part of idiosoma with several pairs of dorso-median setae *dm*. Peripheral setae numerous and much longer than dorsal setae and situated medially and laterally. Leg setae *l’GIV* present. Setae *tc’* and *tc”* of legs II–IV serrate.

*Microhabitat*: Under the ventral and lateral scales of the head, belly, and tail.

*Distribution and host range*: This group is associated with tree lizards of the genus *Liolaemus* (Sauria: Liolaemidae) from Chile.

*Species included*: *Neopterygosoma chilensis* (Fajfer & González-Acuña, 2013), *N. cyanogasteri* (Fajfer & González-Acuña, 2013), *N. formosus* (Fajfer & González-Acuña, 2013), *N*. *levissima* (Fajfer & González-Acuña, 2013), *N*. *ligare* (Fajfer & González-Acuña, 2013), *N*. *ovata* (Fajfer & González-Acuña, 2013), *N. schroederi* n. sp.

***Neopterygosoma chilensis*** **(Fajfer & González-Acuña, 2013)**

Syn. *Pterygosoma chilensis* Fajfer & González-Acuña, 2013

*Type-host*: *Liolaemus chilensis* (Lesson) (Sauria: Liolaemidae).

*Type-locality*: Chile: Río Ñuble (36°52’37”S, 72°05’17”W; 5.xi.2008, coll. D. González-Acuña).

*Type-material*: The holotype female is deposited in the ZISP (Reg. No. ZISP T-Pt-8), 1 female paratype is deposited in the AMU (Reg. No. AMU-PTE5.1).

*Other material examined*: Five females from *Liolaemus chilensis* (NHM no. 1904.10.26.103–106), Chile: Concepción Province, Bío Bío Region, Penco (new locality), 26.x.1904, coll. S. C. Reed. All mite specimens are deposited in the UKSW (Reg. no. UKSW-PTE1.1).

*Records*: Fajfer & González-Acuña (2013: p. 312, figures 13–15); Fajfer (2019: p. 422).

Diagnosis

**Fig. 2:**
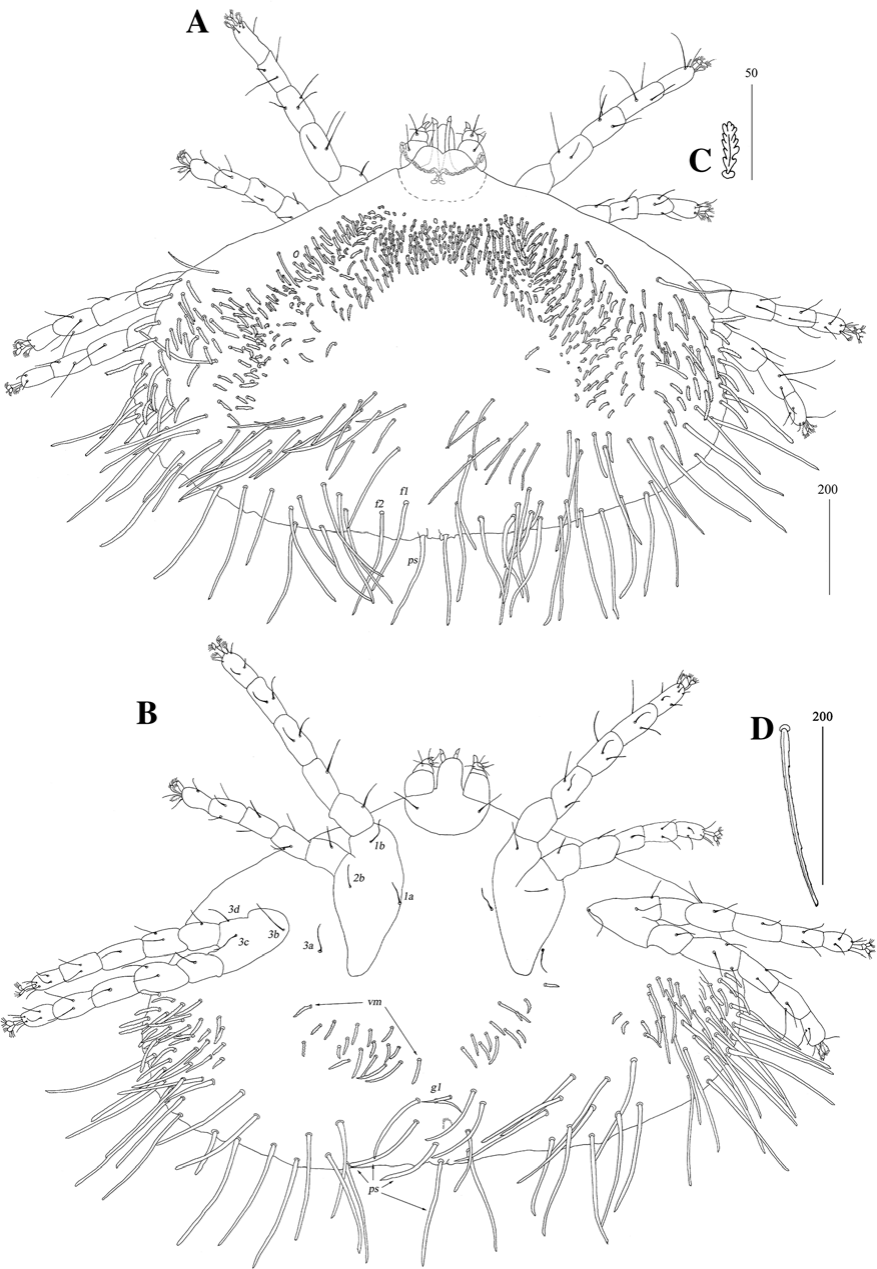
*Neopterygosoma chilensis* (Fajfer & González-Acuña, 2013), female. A, Dorsal view; B, Ventral view; C, Mid-dorsal seta; D, Peripheral seta (after Fajfer & González-Acuña 2013, amended). *Scale-bars*: all in micrometres

**Fig. 3:**
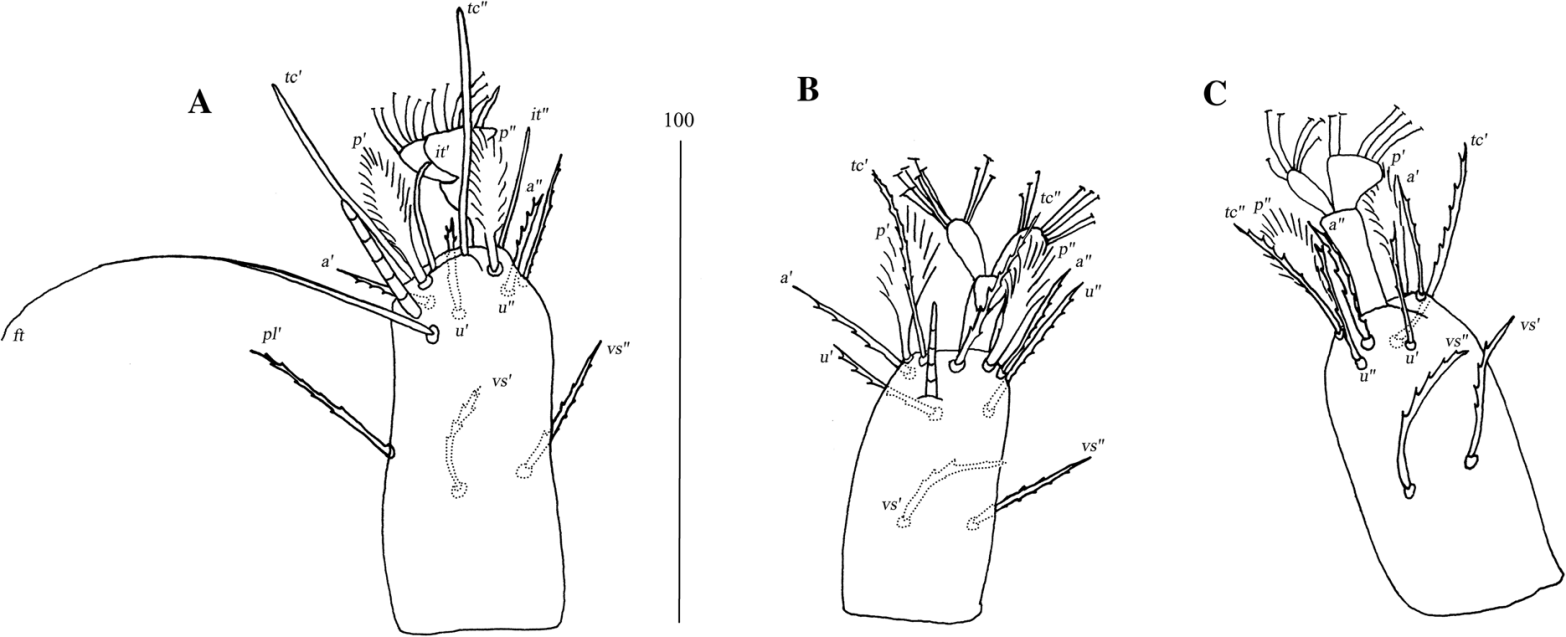
*Neopterygosoma chilensis* (Fajfer & González-Acuña, 2013), female. A, Tarsus I in dorsal view; B, Tarsus II in dorsal view; C, Tarsus IV in ventral view (after Fajfer & González-Acuña, 2013, amended). *Scale-bars*: all in micrometres

*Female* [Based on the holotype, 1 paratype and 5 non-type females; Figs. 2, 3.] *Gnathosoma.* Fixed cheliceral digit short and with spinous process. Setae *dF* and *dG* serrate. *Idiosoma* 830−965 long, 1,280−1,520 wide. Antero-lateral part of dorsum with *c.*68 plumose setae grouped in cluster; setae increasing in length from anterior to posterior part of this cluster. Lateral to this cluster situated *c.*170–200 pairs of plumose setae. Among them 1 very long seta, 120 long, present on each lateral margin. About 20 pairs of serrate setae located anterior to each side of pseudanal area present. Lateral parts of idiosoma with slightly apically expanded setae. Peripheral series represented by 13 pairs of apically expanded setae. Venter with 12–18 pairs of plumose setae *vm* located anterior to genital area. Lateral parts covered with 39–42 pairs of setae increasing in length from ventral to lateral part of idiosoma. Shorter setae plumose, longer setae serrate at distal part. Peripheral series represented by 10–13 pairs of slightly serrate setae. Genital series represented by slightly serrate setae *g1* and 5 pairs of pseudanal setae *ps*. *Legs* chaetotaxy is provided in Table 1. Coxal setae *3a* filiform. Setation of tarsi I-IV is given in Table 2. Setae *tc’* and *tc*” of legs II-IV serrate. All setae *a’* and *a”* smooth, *u’* and *u”* pectinate, *vs’* and *vs”* bipectinate.

**Table 1:** Summary of leg chaetotaxy of the tibiae-coxae I-IV of the species analysed here

Species	Tibia	Genu	Femur	Trochanter	Coxae	No. of setae
*N. ovata*, *N. levissima*, *N. cyanogasteri*, *N. ligare*	5-5-5-5	5-4-3-3	5-5-3-3	1-1-1-1	2-1-4-0	62
*N. formosus*	5-5-5-5	4-4-3-3	5-4-3-2	1-1-1-1	2-1-4-0	59
*N. chilensis*, *N. schroederi*	5-5-5-5	5-4-3-3	5-4-3-3	1-1-1-1	2-1-4-0	61
*N. patagonica*	5-4(5)-5-5	5-4-3-2	5-4-3-2	1-1-1-1	2-1-4-0	58 or 59

**Table 2:** Tarsal chaetotaxy of the genus *Neopterygosoma*

Legs	Setae	No. of setae
I	*ft*, *tc*′(*ζ*), *tc*″(*ζ*), *p*′, *p*″, *a*′, *a*″, *it*′(*ζ*), *it*″(*ζ*), ***u*′**, ***u*″**, ***vs*′**, ***vs*″**, *pl*′	14 (+ *solenidion*)
II	*tc*′, *tc*″, *p*′, *p*″, *a*′, *a*″, ***u*′**, ***u*″**, ***vs*′**, ***vs*″**	10 (+ *solenidion*)
III	*tc*′, *tc*″, *p*′, *p*″, *a*′, *a*″, ***u*′**, ***u*″**, ***vs*′**, ***vs*″**	10
IV	*tc*′, *tc*″, *p*′, *p*″, *a*′, *a*″, ***u***′, ***u*″**, ***vs*′**, ***vs*″**	10

Designation: ventral setae marked as bold, euphatidial setae in parentheses marked as *ζ*.

**Table 3:** Scale-mites of the genus *Neopterygosoma* with their host associations (Reptilia: Sauria)

*Neopterygosoma*	Sauria: Liolaemidae	South America	Main reference
*N. chilensis* Fajfer & González-Acuña, 2013	*Liolaemus chilensis* (Lesson)	Chile	Fajfer & González-Acuña (2013); this study
*N. cyanogasteri* Fajfer & González-Acuña, 2013	*Liolaemus cyanogasteri* (Duméril & Bibron)	Chile	Fajfer & González-Acuña (2013)
*N. formosus* Fajfer & González-Acuña, 2013	*Liolaemus pictus* (Duméril & Bibron)	Chile	Fajfer & González-Acuña (2013)
*N. levissima* Fajfer & González-Acuña, 2013	*Liolaemus pictus* (Duméril & Bibron)	Chile	Fajfer & González-Acuña (2013)
*N. ligare* Fajfer & González-Acuña, 2013	*Liolaemus pictus* (Duméril & Bibron)	Chile	Fajfer & González-Acuña (2013)
*N. ovata* Fajfer & González-Acuña, 2013	*Liolaemus pictus* (Duméril & Bibron)	Chile	Fajfer & González-Acuña (2013)
*N. patagonica* Dittmar de la Cruz, Morando & Avila, 2004	*Liolaemus austromendocinus* Cei	Argentina	Dittmar de la Cruz et al. (2004); Fajfer (2014); this study
	*Liolaemus bibronii* Bell	Argentina	Dittmar de la Cruz et al. (2004); Fajfer (2014)
	*Liolaemus buergeri* Werner	Argentina	Dittmar de la Cruz et al. (2004); Fajfer (2014)
	*Liolaemus elongatus* Koslowsky	Argentina	Dittmar de la Cruz et al. (2004); Fajfer (2014)
	*Liolaemus gracilis* (Bell)	Argentina	Dittmar de la Cruz et al. (2004); Fajfer (2014)
	*Liolaemus pethrophilus* Donoso-Barros Cei	Argentina	Dittmar de la Cruz et al. (2004); Fajfer (2014); this study
	*Liolaemus rothi* Koslowsky	Argentina	Fajfer (2014); this study
*N. schroederi* n. sp.	*Liolaemus schroederi* Müller & Hellmich	Chile	This study

*Male*. Unknown.

### Remarks

In the original description of the species (Fajfer & González-Acuña, 2013) some inaccuracies are mentioned, i.e. eyes are marked as absent but they are present, setae *a’* and *a”* of tarsi I are marked as eupathidia but they are simple, setae *it'* and *it"* are marked as simple but they are eupathidia.

***Neopterygosoma cyanogasteri*** **(Fajfer & González-Acuña, 2013)**

Syn. *Pterygosoma cyanogasteri* Fajfer & González-Acuña, 2013

*Type-host*: *Liolaemus cyanogaster* Duméril & Bibron (Sauria: Liolaemidae).

*Type-locality*: Chile (ZMUC-R37901) (19.vi.1886, coll. Mr Jessen).

*Type-material*: The holotype female is deposited in the ZMUC (Reg. no. ZMUC-R37901). Paratypes: 3 females in the ZMUC (Reg. no. ZMUC-R37901), 3 paratypes in the AMU (Reg. no. AMU-PTE6.1) and 3 paratypes in the ZISP (Reg no. ZISP T-Pt-9).

*Records:* Fajfer & González-Acuña (2013: p. 316, figures 16–20); Fajfer (2019: p. 422)

Diagnosis

**Fig. 4:**
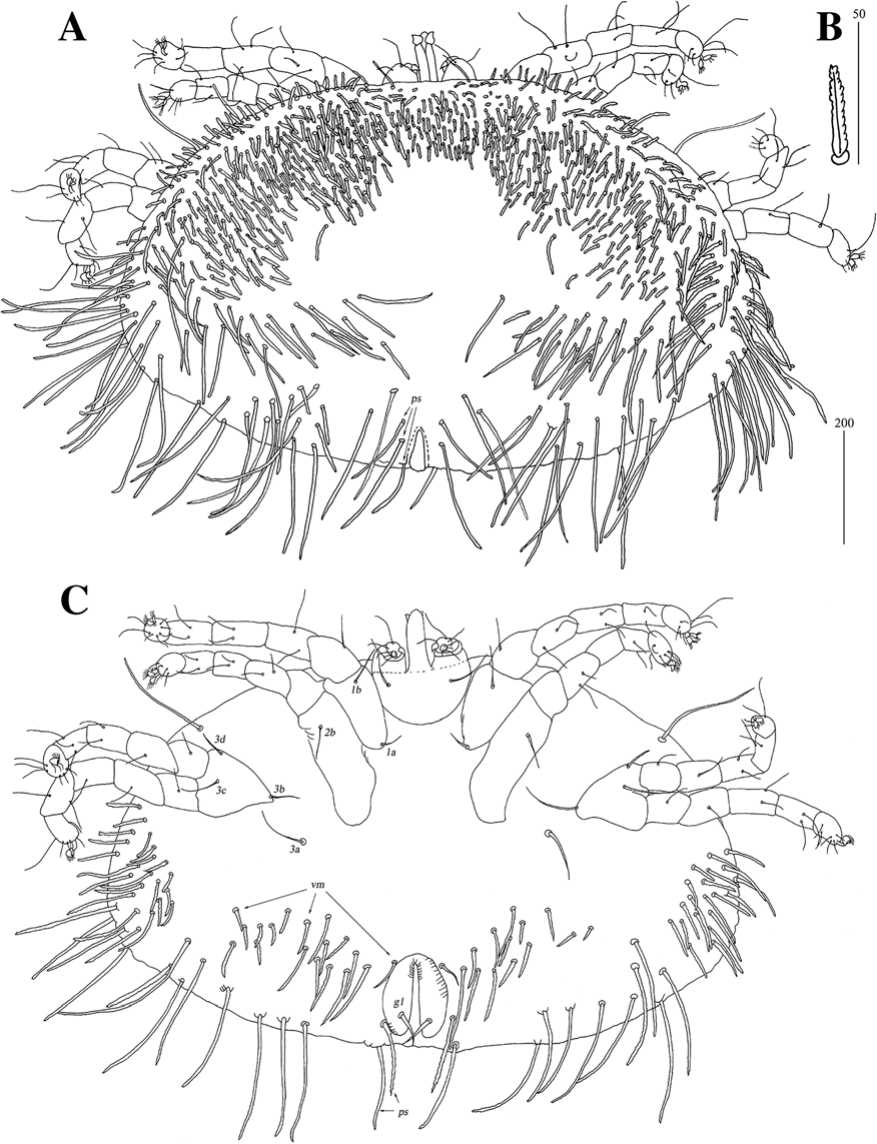
*Neopterygosoma cyanogasteri* (Fajfer & González-Acuña, 2013), female. A, Dorsal view; B, Antero-dorsal seta; C, Ventral view (after Fajfer & González-Acuña, 2013, amended). *Scale-bars*: all in micrometres

**Fig. 5:**
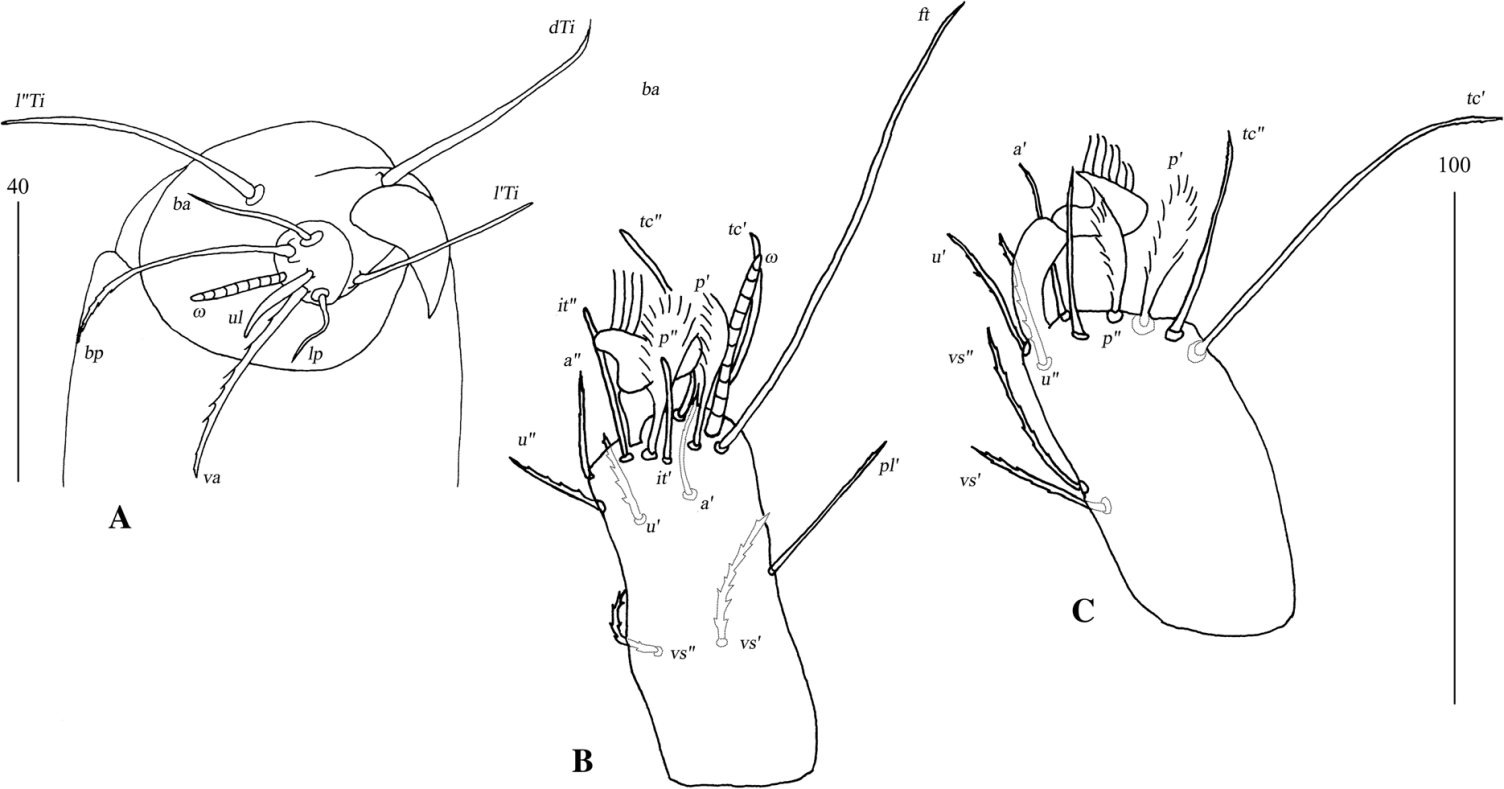
*Neopterygosoma cyanogasteri* (Fajfer & González-Acuña, 2013), female. A, Palpal tarsus and tibia, ventral view; B, Tarsus I, dorsal view; C, Tarsus IV, lateral view (after Fajfer & González-Acuña, 2013, amended). *Scale-bars*: all in micrometres

*Female* [Based on the holotype and 10 paratypes; Figs. 4, 5.] *Gnathosoma*. Swollen, proximal part of cheliceral base longer than slender, distal part. Fixed cheliceral digit with spinous process. Seta *dF* and *dG* serrate. Subcapitular setae *n* serrate. *Idiosoma* 515–680 long, 965−1,150 wide. Antero-medial part of dorsum with *c.*60 plumose setae grouped in cluster. Lateral to this cluster *c.*230 plumose setae present on each side. Among them 1 longer seta, 95 long, on each lateral margin. Plumose setae 17–21 pairs, located anterior to each side of pseudanal area. Postero-lateral part of idiosoma with 15 pairs of apically serrate setae. Peripheral series represented by 12–13 pairs of distally serrate setae. Venter with 14–18 pairs of setae *vm*, located anterior to genital area. All setae *vm* serrate, except for 1 pair of filiform setae situated laterally to genital area. Peripheral part of body with 20–25 pairs of postero-lateral setae and 9–10 pairs of peripheral slightly serrate setae situated posteriorly. Genital series represented by smooth setae *g1* and 5 pairs of pseudanal setae *ps*. *Legs* chaetotaxy as in *ovata* group (Table 1). Coxal setae *3a* slightly serrate. Setation of tarsi I-IV as in Table 2. Setae *tc’* and *tc”* of legs II-IV slender and serrate only at distal margins.

*Male*. Unknown.

### Remarks

In the original description of the species (Fajfer & González-Acuña, 2013) some inaccuracies are mentioned, i.e. eyes are marked as absent but they are present, setae *a’* and *a”* of tarsi I are marked as eupathidia but they are simple, setae *it'* and *it" *are marked as simple but they are eupathidia.

***Neopterygosoma formosus*** **(Fajfer & González-Acuña, 2013)**

Syn. *Pterygosoma formosus* Fajfer & González-Acuña, 2013

*Type-host*: *Liolaemus pictus* (Duméril & Bibron) (Sauria: Liolaemidae).

*Type-locality*: Chile: Arauco Province, Isla Mocha (38°36’32”S, 73°89’87”W (13.xii.2008, coll. D. González-Acuña).

*Type material*: The holotype female is deposited in the ZISP (Reg no. ZISP T-Pt-5); 1 paratype female is deposited in the AMU (Reg. no. AMU-PTE2.1).

*Records*: Fajfer & González-Acuña (2013: p. 305, figures 4–6); Fajfer (2019: p. 422).

Diagnosis

**Fig. 6:**
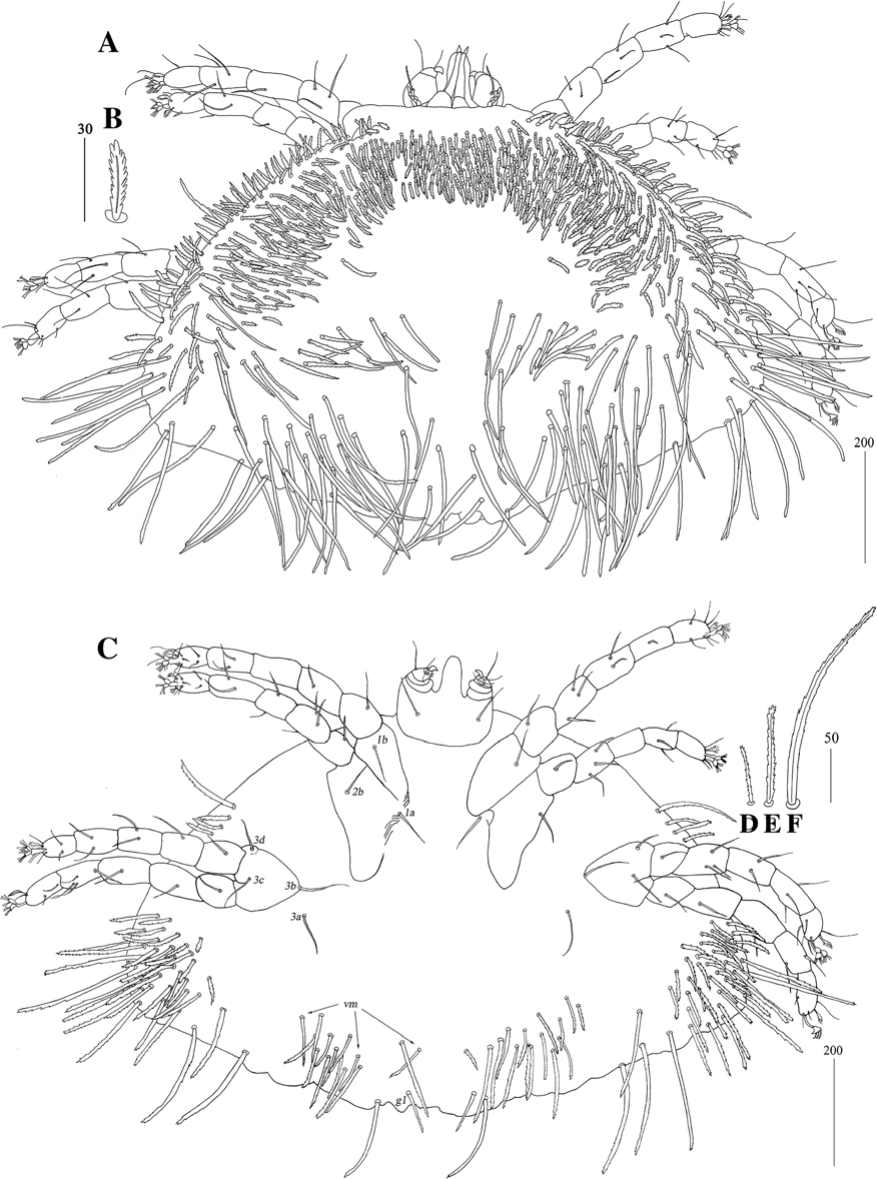
*Neopterygosoma formosus* (Fajfer & González-Acuña, 2013), female. A, Dorsal view; B, Antero-dorsal seta; C, Ventral view; D, Coxal seta*3b*; E, Postero-lateral seta; F, Peripheral seta (after Fajfer & González-Acuña, 2013, amended). *Scale-bars*: all in micrometres

**Fig. 7:**
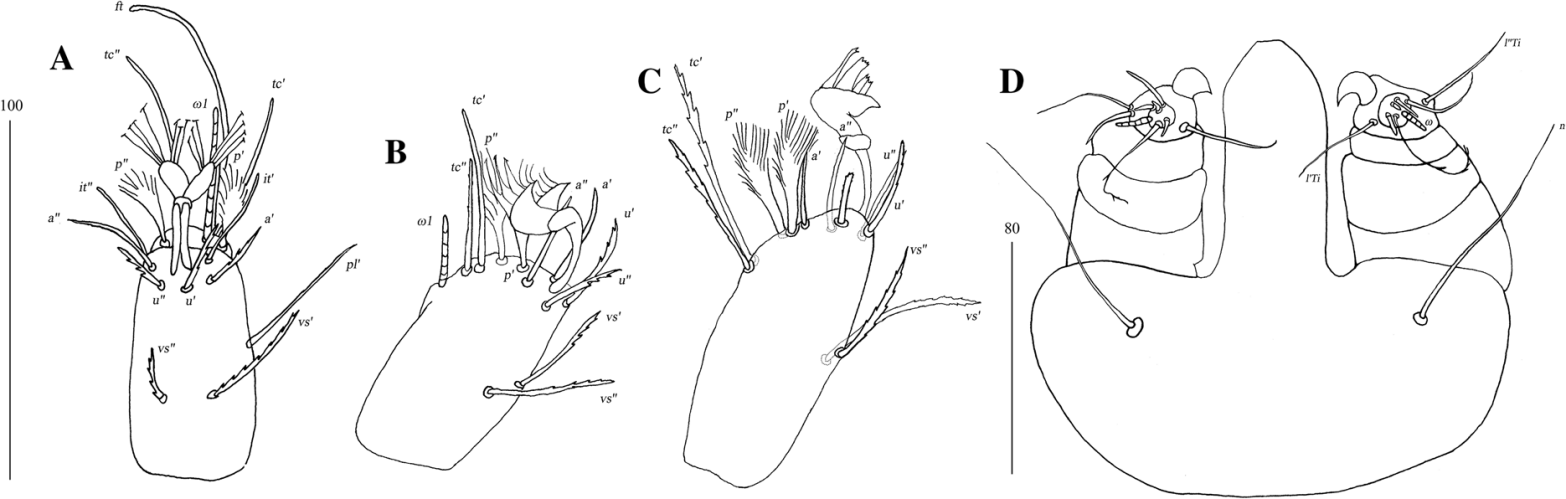
*Pterygosoma formosus* (Fajfer & González-Acuña, 2013), female. A, Tarsus I, ventral view; B, Tarsus II, lateral view; C, Tarsus IV, lateral view, D, Gnathosoma, ventral view (after Fajfer & González-Acuña, 2013, amended). *Scale-bars*: all in micrometres

*Female* [Based on the holotype and 1 paratype; Figs. 6, 7.] *Gnathosoma*. Swollen, proximal part of cheliceral base and slender, distal part equal in length. Palpal femur and genu with serrate dorsal seta each. Subcapitular setae *n* smooth. *Idiosoma* 650–735 long, 1,015–1,165 wide. Antero-medial part of dorsum with *c.*110 plumose setae grouped in cluster. Lateral to this cluster, *c.*220–230 plumose setae present on each side. Among them, 1 very long seta, 135 long, present on each lateral margin. Anterior to each side of pseudanal area, distinctly plumose setae, *c.*16 pairs, present. Postero-lateral part of idiosoma with short serrate setae and long setae serrate only on tips. Posterior parts of idiosoma with 21 or 22 pairs of setae slightly expanded apically. Venter with 13 or 14 pairs of setae *vm* located anterior to genital area. Peripheral part of body with 32–36 pairs of postero-lateral setae. Genital series represented by slightly serrate setae *g1* and 3 pairs of pseudanal setae *ps*. *Legs* chaetotaxy is given in Table 1. Coxal setae *3a* slightly serrate. Setation of tarsi I-IV as in Table 2, but additional seta present on tarsus IV.

*Male*. Unknown.

### Remarks

In the original description of the species (Fajfer & González-Acuña, 2013) some inaccuracies are mentioned, i.e. eyes are marked as absent but they are present, setae *a’* and *a”* of tarsi I are marked as eupathidia but they are simple, setae *it'* and *it"* are marked as simple but they are eupathidia.

***Neopterygosoma levissima*** **(Fajfer & González-Acuña, 2013)**

Syn. *Pterygosoma levissima* Fajfer & González-Acuña, 2013

*Type-host*: *Liolaemus pictus* (Duméril & Bibron) (Sauria: Liolaemidae).

*Type-locality*: Chile: Arauco Province, Isla Mocha (38°36’32”S, 73°89’87”W; 13.xii.2008, coll. D. González-Acuña).

*Type-material*: The holotype female is deposited in the ZISP (Reg. No. ZISP T-Pt-7), 1 paratype female is deposited in the AMU (Reg. No. AMU-PTE4.1).

*Records*: Fajfer & González-Acuña (2013: p. 311 figures 10–12); Fajfer (2019: p. 422)

Diagnosis

**Fig. 8:**
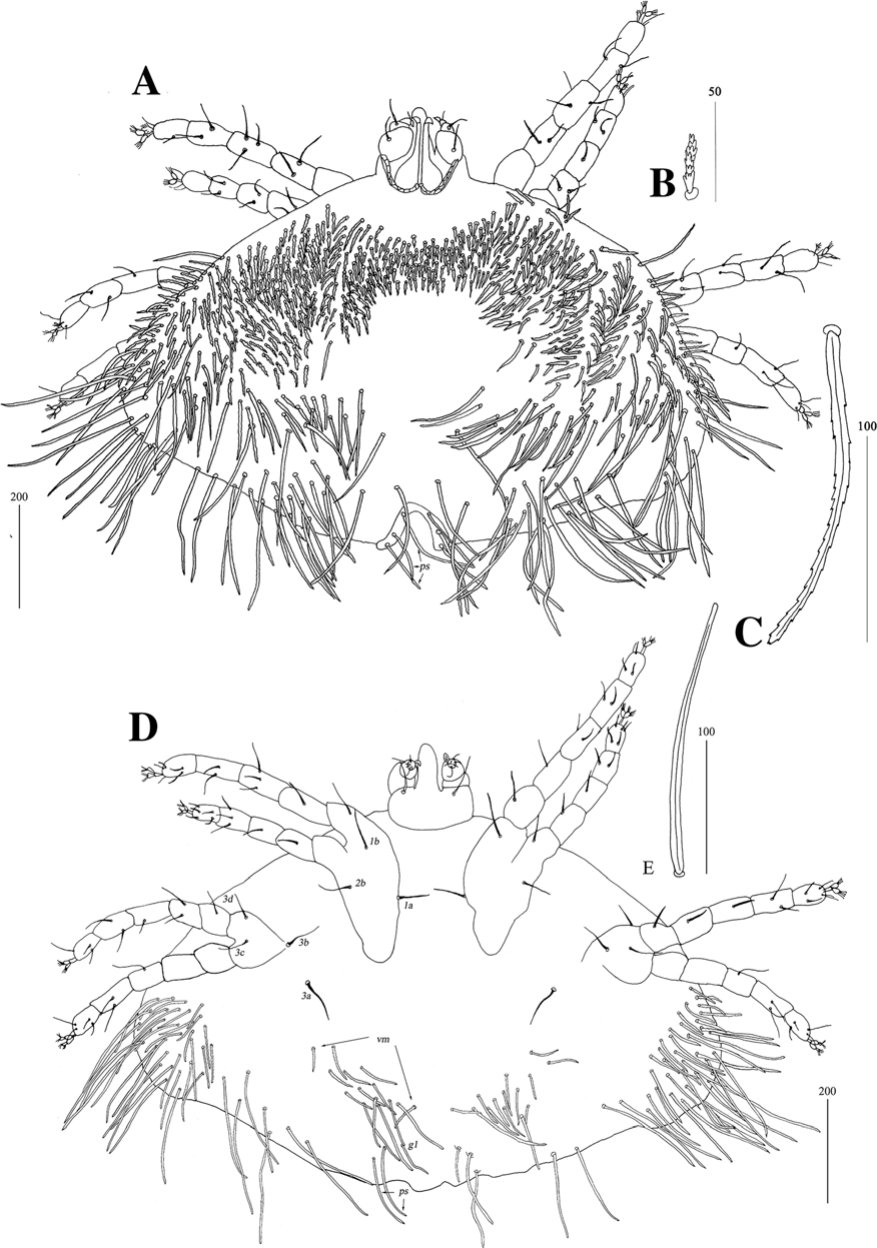
*Neopterygosoma levissima* (Fajfer & González-Acuña, 2013), female. A, Dorsal view; B, Antero-dorsal seta; C, Peripheral seta; D, Ventral view; E, Peripheral seta (after Fajfer & González-Acuña, 2013, amended). *Scale-bars*: all in micrometres

**Fig. 9:**
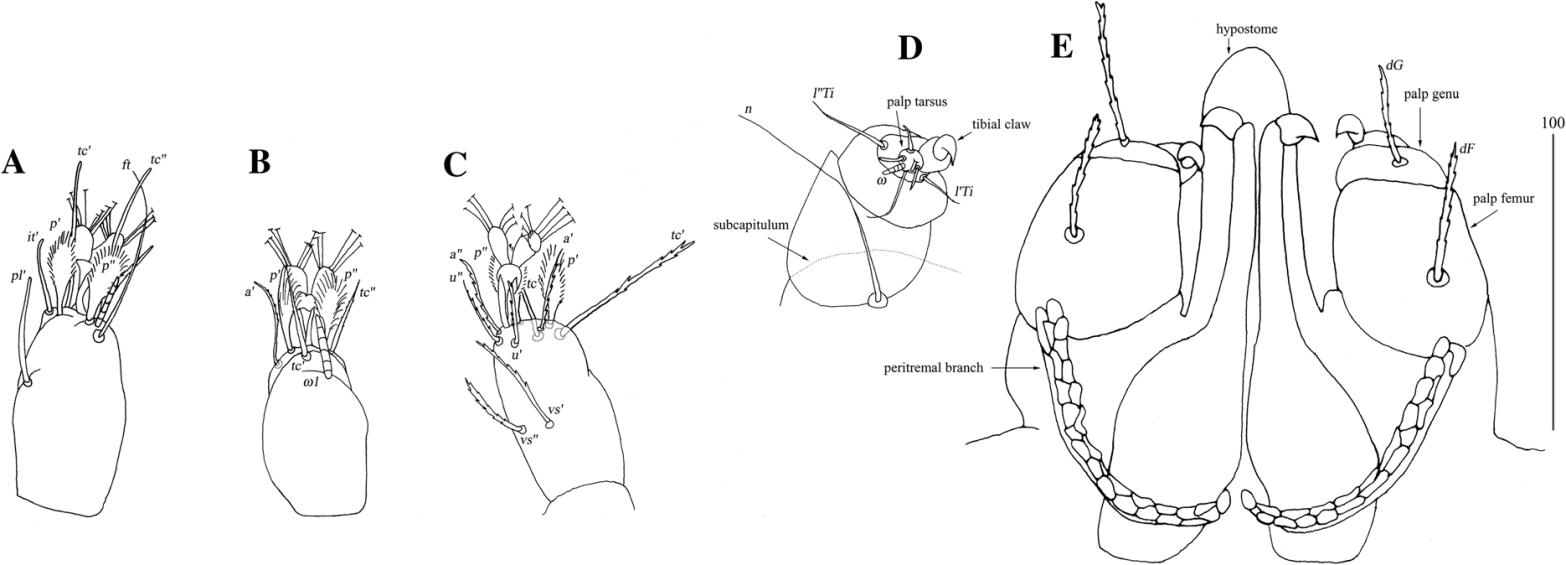
*Neopterygosoma levissima* (Fajfer & González-Acuña, 2013), female. A, Tarsus I, dorsal view; B, Tarsus II, dorsal view; C, Tarsus IV, ventral view; D, Palpal tarsus, ventral view; E, Gnathosoma, dorsal view (after Fajfer & González-Acuña, 2013, amended). *Scale-bars*: all in micrometres

*Female* [Based on the holotype and 1 female paratype; Figs. 8, 9.] *Gnathosoma*. Swollen, proximal part of cheliceral base and slender, distal part equal in length. Palpal femur and genu with serrate dorsal seta each. Subcapitular setae *n* smooth. *Idiosoma* 605–735 long, 1,065–1,080 wide. Dorsum. Antero-medial part with *c.*80 plumose setae grouped in cluster. Lateral to this cluster *c.*210–230 plumose setae present on each side. Among them, 1 very long seta, 135 long, present on each lateral margin. Finely serrate setae, *c.*20 pairs, located anterior to each side of pseudanal area. Postero-lateral part of idiosoma with *c.*20–25 pairs of setae, only slightly serrate on tips. Peripheral series represented by 21–23 pairs of tapered setae, discernibly serrate distally. Venter with 15 pairs of setae *vm* located anterior to genital area. All setae *vm* serrate, except for 1 pair of filiform setae located near to genital region. Peripheral part of body with 36 pairs of postero-lateral setae: short setae serrate, long setae smooth. Peripheral series represented by 6–13 pairs of smooth setae weakly serrate apically. Genital series represented by slightly serrate setae *g1* and 5 pairs of pseudanal setae *ps*. *Legs* chaetotaxy as in *ovata* group (Table 1). Coxal setae *3a* filiform. Setation of tarsi I-IV as in Table 2.

*Male*. Unknown.

### Remarks

In the original description of the species (Fajfer & González-Acuña, 2013) some inaccuracies are mentioned, i.e. eyes are marked as absent but they are present, setae *a’* and *a”* of tarsi I are marked as eupathidia but they are simple, setae *it'* and *it"* are marked as simple but they are eupathidia.

***Neopterygosoma ligare*** **(Fajfer & González-Acuña, 2013)**

Syn. *Pterygosoma ligare* Fajfer & González-Acuña, 2013

*Type-host*: *Liolaemus pictus* (Duméril & Bibron) (Sauria: Liolaemidae).

*Type-locality*: Chile: Arauco Province, Isla Mocha (38°36’32”S, 73°89’87”W; 13.xii.2008, coll. D. González-Acuña).

*Type-material*: The holotype female is deposited in the ZISP (Reg No. ZISP T-Pt-4); 3 paratype females are deposited in the ZISP (Reg No. ZISP T-Pt-4) and AMU (Reg. No. AMU-PTE1.1)

*Records*: Fajfer & González-Acuña (2013: p. 303, figures 1–3); Fajfer (2019: p. 422).

Diagnosis

**Fig. 10:**
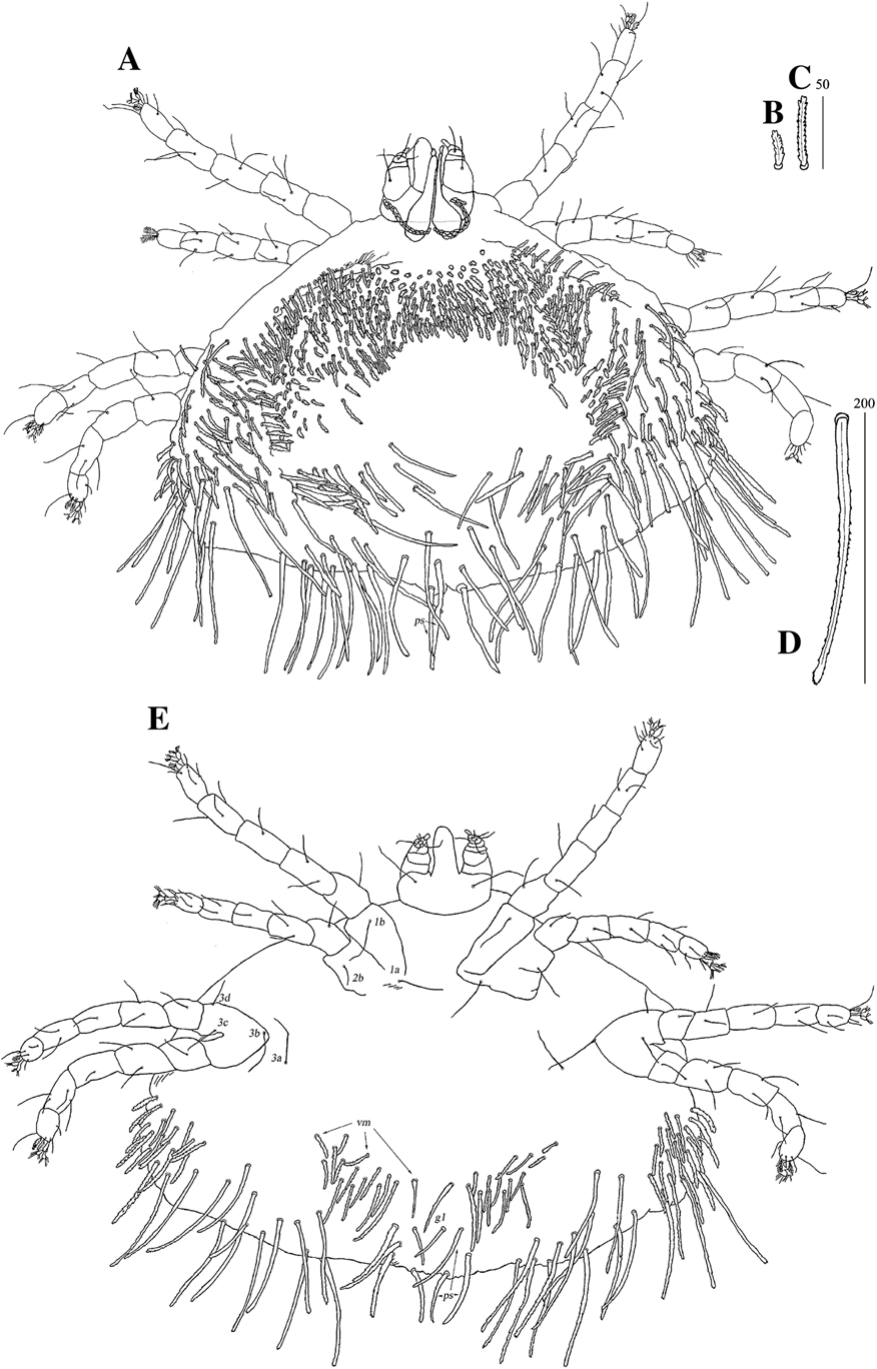
*Neopterygosoma ligare* (Fajfer & González-Acuña, 2013), female. A, Dorsal view; B, Antero-dorsal seta; C, Medio-lateral seta; D, Peripheral seta; E, Ventral view (after Fajfer & González-Acuña, 2013, amended). *Scale-bars*: all in micrometres

**Fig. 11:**
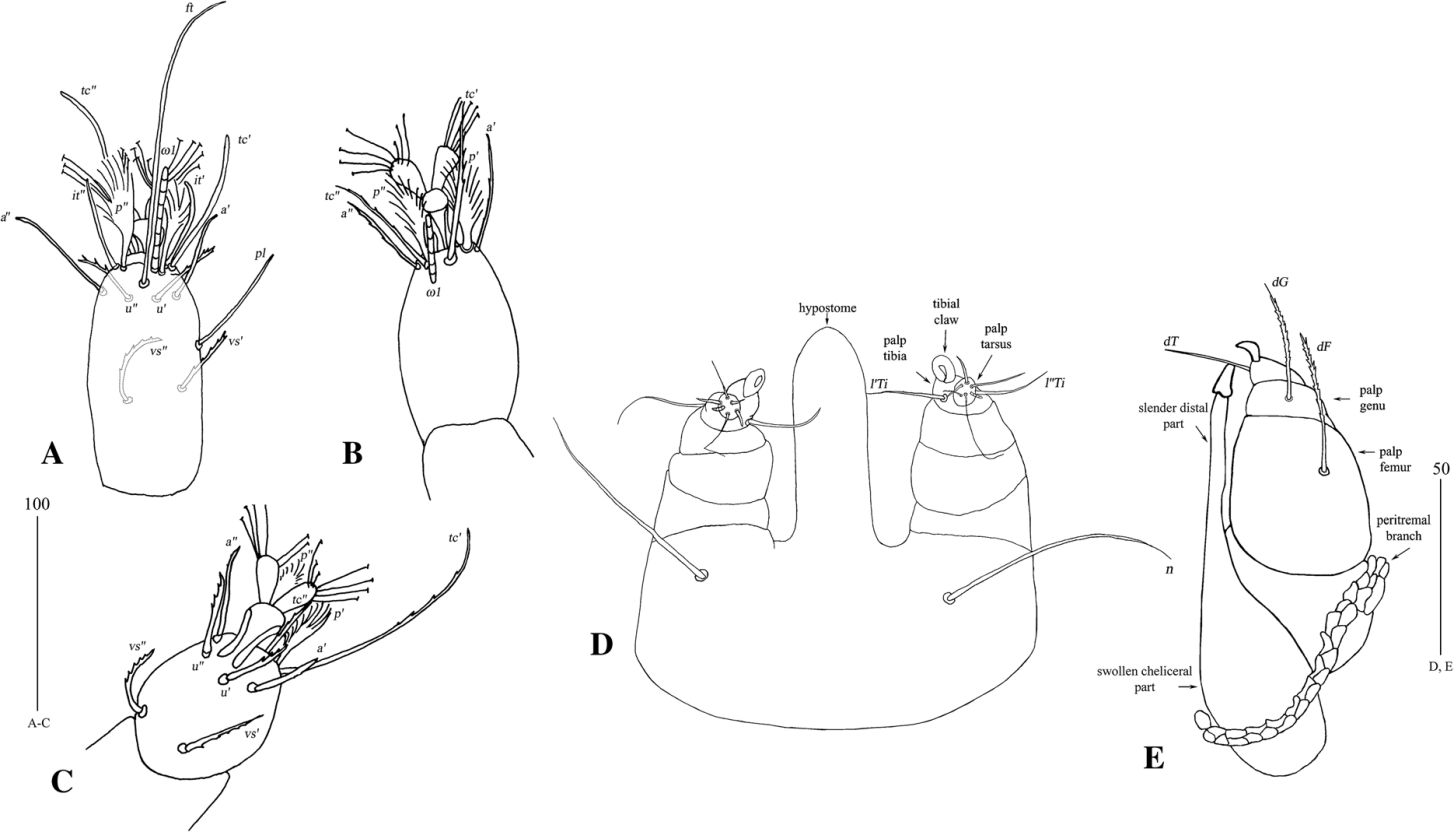
*Neopterygosoma ligare* (Fajfer & González-Acuña, 2013), female. A, Tarsus I, dorsal view; B, Tarsus II, dorsal view; C, Tarsus IV, ventral view; D, Gnathosoma, ventral view; E, Part of gnathosoma, dorsal view (after Fajfer & González-Acuña, 2013, amended). *Scale-bars*: all in micrometres

*Female* [Based on the holotype and 3 paratypes; Figs. 10, 11.] *Gnathosoma*. Swollen, proximal part of cheliceral base shorter than slender, distal part. Palpal femur and genu with serrate dorsal seta each. Subcapitular setae *n* smooth. *Idiosoma*, 655*–*745 long, 1,050*–*1,135 wide. Dorsum with anterior-mid setal cluster with 86 plumose setae that increase in length from anterior to posterior parts of this cluster. Lateral to this cluster, *c.*158–160 setae present on each side. Among them, 1 very long seta, 120 long, present on each lateral margin of dorsum. Plumose setae 15 or 16 pairs, located anterior to each side of pseudanal area. Lateral parts of idiosoma with 24 or 25 pairs of slightly apically expanded setae. Peripheral series represented by 12 pairs of setae slightly serrate distally. Venter with 15 pairs of setae *vm* located anterior to genital area. Peripheral part of body with 19 pairs of postero-lateral plumose setae and 13 pairs of peripheral weakly serrate setae situated posteriorly. Genital series represented by slightly serrate setae *g1* and 5 pairs of pseudanal setae *ps*. *Legs* chaetotaxy as in *ovata* group (Table 1). Coxal setae *3a* slightly serrate. Ventral setae of leg I serrate only at distal tip. Setation of tarsi I-IV as in Table 2.

*Male*. Unknown.

### Remarks

In the original description of the species (Fajfer & González-Acuña, 2013) some inaccuracies are mentioned, i.e. eyes are marked as absent but they are present, setae *a’* and *a”* of tarsi I are marked as eupathidia but they are simple, setae *it'* and *it"* are marked as simple but they are eupathidia.

***Neopterygosoma ovata*** **(Fajfer & González-Acuña, 2013)**

Syn. *Pterygosoma ovata* Fajfer & González-Acuña, 2013

*Type-host*: *Liolaemus pictus* (Duméril & Bibron) (Sauria: Liolaemidae).

*Type-locality*: Chile: Arauco Province, Isla Mocha (38°36’32”S, 73°89’87”W; 13.xii.2008, coll. D. González-Acuña).

*Type-material*: The holotype female is deposited in the ZISP (Reg. No. ZISP T-Pt-6), one paratype female is deposited in the AMU (Reg. No. AMU-PTE3.1).

*Records*: Fajfer & González-Acuña (2013: p. 308, figures 7–9).

*Neopterygosoma ovata*, Fajfer 2019: 422.

Diagnosis

**Fig. 12:**
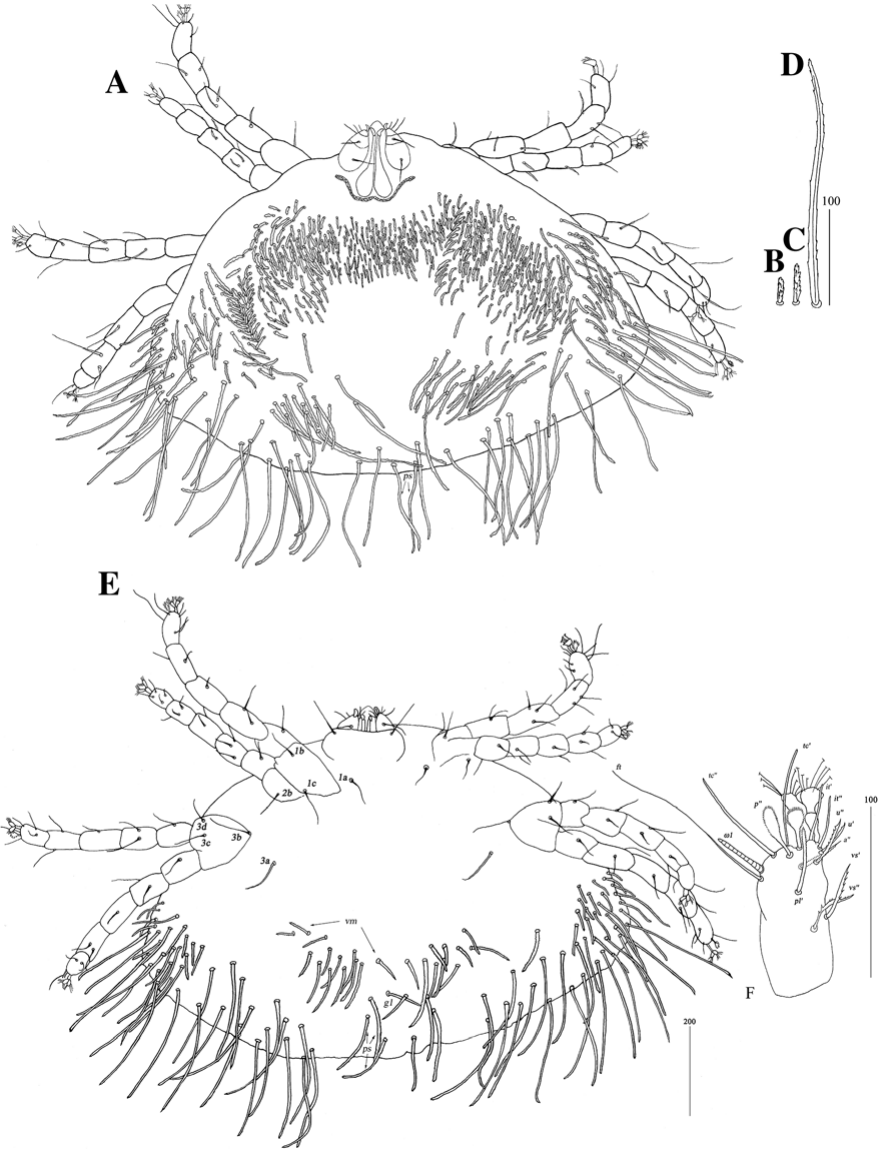
*Neopterygosoma ovata* (Fajfer & González-Acuña, 2013), female. A, Dorsal view; B, Antero-dorsal seta; C, Mid-dorsal seta; D, Peripheral seta; E, Ventral view; F, Tarsus I in ventro-lateral view (after Fajfer & González-Acuña, 2013, amended). *Scale-bars*: all in micrometres

*Female* [Based on the holotype and 1 paratype; Fig. 12.] *Gnathosoma*. Swollen, proximal part of cheliceral base shorter than slender distal part. Palpal femur with serrate seta *dF*, palpal genu with seta *dG* serrate only at distal part. Subcapitular setae *n* smooth. *Idiosoma* 620–650 long, 1,080–1,090 wide. Dorsum. Antero-medial part with *c.*90 setae grouped in cluster. Lateral to this cluster, *c.*150 setae present on each side. Among these setae 2–3 longer setae, 85 long, present on each lateral margin of dorsum. Plumose setae, 17–21 pairs, located anterior to each side of pseudanal area. Postero-lateral part of idiosoma with setae slightly expanded on tips. Peripheral series represented by 12 or 13 pairs of setae, discernibly serrate only distally. Venter with 10–13 pairs of finely serrate setae *vm* located anterior to genital area. Peripheral part of idiosoma with 24–27 pairs of postero-lateral slightly plumose setae and 15 pairs of weakly serrate setae situated posteriorly. Genital series represented by slightly serrate setae *g1* and 5 pairs of pseudanal setae *ps*. *Legs* chaetotaxy as in Table 1, coxae with additional setae *1a*. Setae *1a* and *3a* situated on intercoxal area. Coxal setae *3a* slightly serrate. Ventral setae of legs I almost filiform, with barely discernible serration. Setation of tarsi I-IV as in Table 2.

*Male*. Unknown.

### Remarks

In the original description of the species (Fajfer & González-Acuña, 2013) some inaccuracies are mentioned, i.e. eyes are marked as absent but they are present, setae *a’* and *a”* of tarsi I are marked as eupathidia but they are simple, setae *it'* and *it"* are marked as simple but they are eupathidia.

***Neopterygosoma schroederi*** **n. sp.**

*Type-host*: *Liolaemus schroederi* Müller & Hellmich (Sauria: Liolaemidae).

*Type-locality*: Chile, Maule region, Curicó Province, Curicó (Reg. No. 54/1933) (20.i.1933, coll. W. Schroeder).

*Type-material*: The holotype female is deposited in the UKSW (Reg. No. UKSW-PTE3.1). Paratypes: 3 paratypes (1 deutonymph and 2 protonymphs) are deposited in the UKSW (Reg. No. UKSW-PTE3.1) and 2 paratypes (1 deutonymph and 3 protonymphs) are deposited in the ZSM (Reg. No. 54/1933).

*ZooBank registration*: To comply with the regulations set out in Article 8.5 of the amended 2012 version of the *International Code of Zoological Nomenclature* (ICZN, 2012), details of the new species have been submitted to ZooBank. The Life Science Identifier (LSID) for *Neopterygosoma schroederi* n. sp. is urn:lsid:zoobank.org:act:2DA68446-7D51-4329-9709-5D2C31CB63DD.

*Etymology*: The new species is named after William C. Schroeder (1894–1977), U.S. oceanographer and ichthyologist.

### Description

**Fig. 13:**
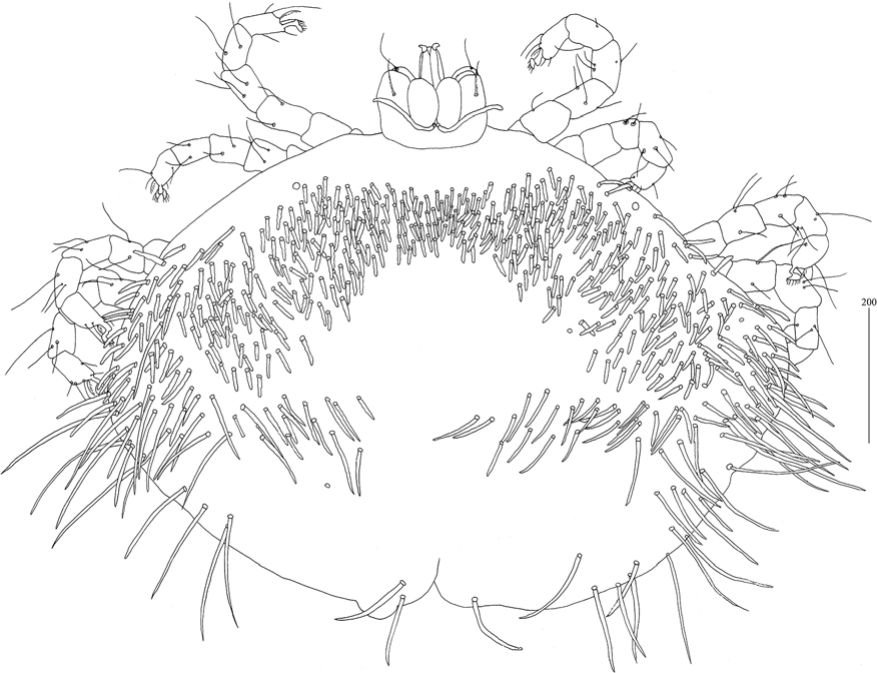
*Neopterygosoma schroederi* n. sp. Female, dorsal view. *Scale-bars*: all in micrometres

**Fig. 14:**
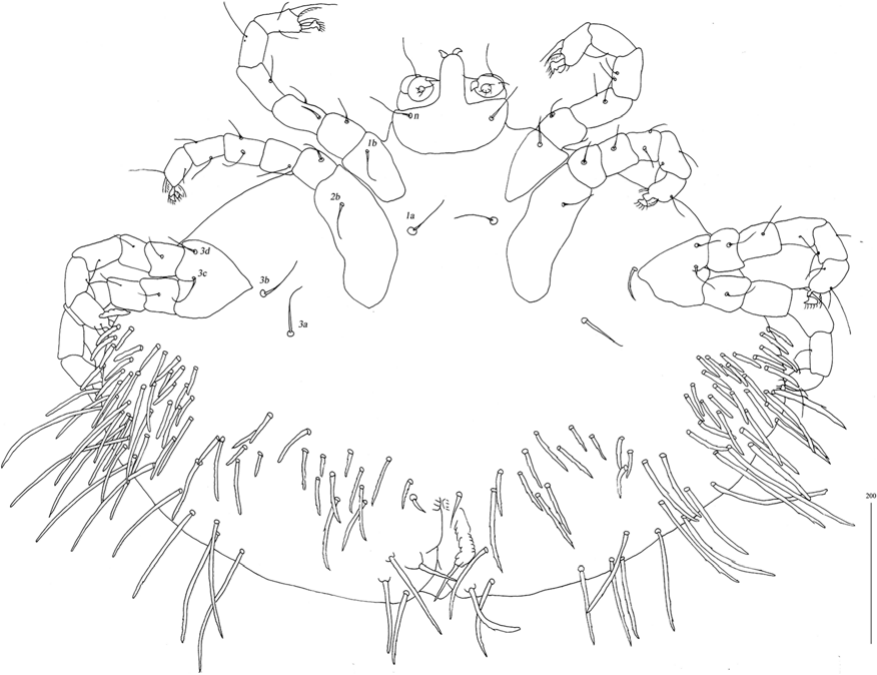
*Neopterygosoma schroederi* n. sp. Female, ventral view. *Scale-bars*: all in micrometres

**Fig. 15:**
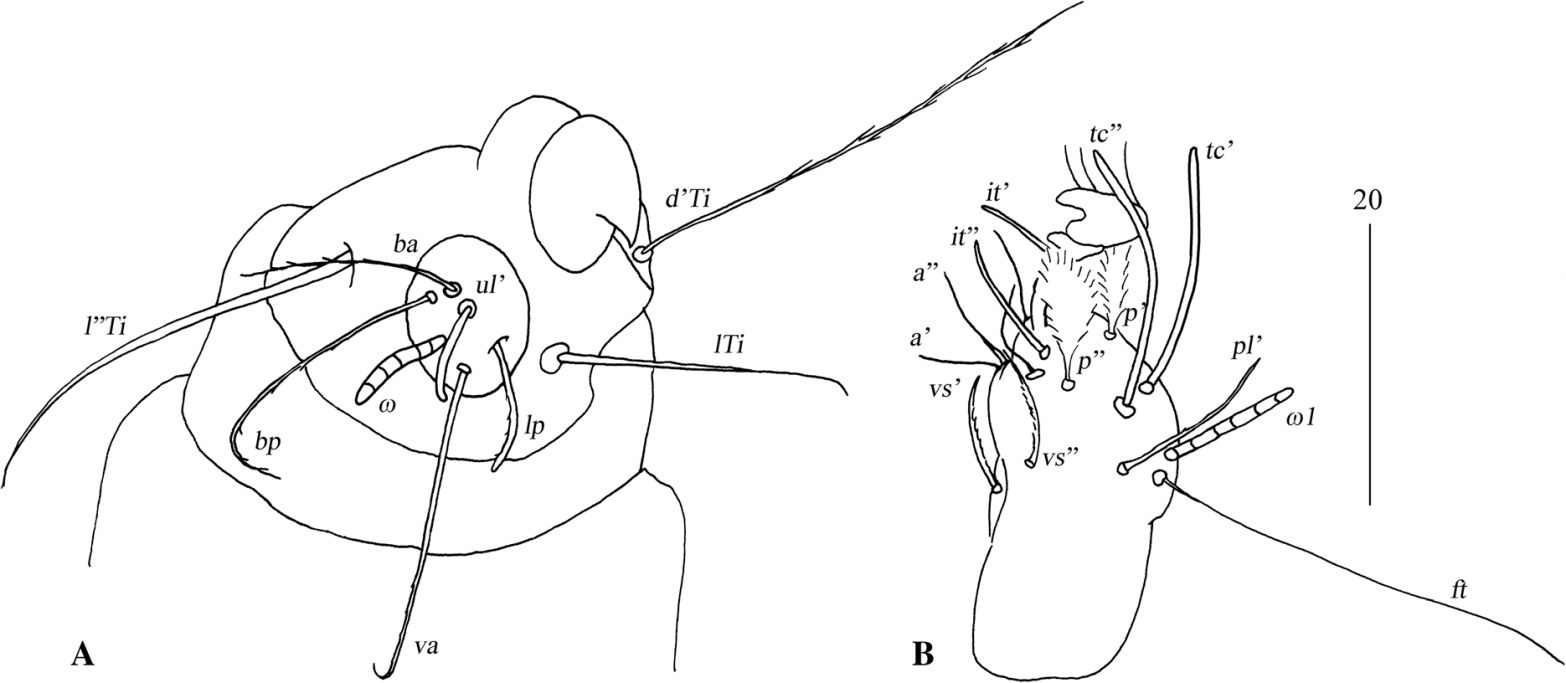
*Neopterygosoma schroederi* n. sp. Female. A, Palp tarsus and tibia, ventral view; B, Tarsi I, lateral view. *Scale-bars*: all in micrometres

**Fig. 16:**
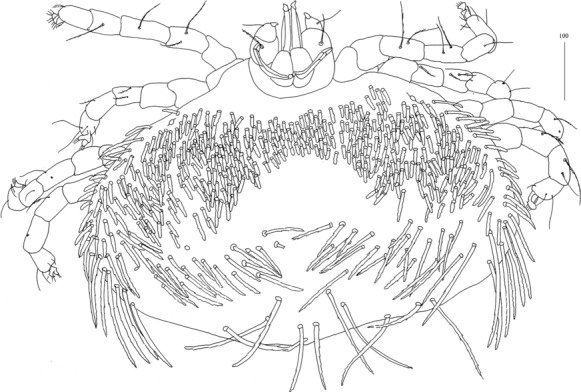
*Neopterygosoma schroederi* n. sp. Deutonymph, dorsal view. *Scale-bars*: all in micrometres

**Fig. 17:**
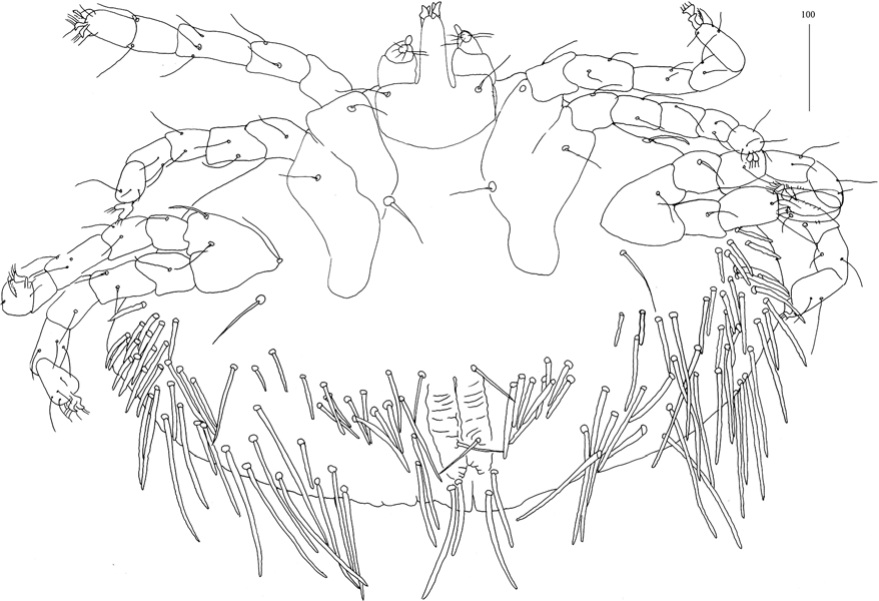
*Neopterygosoma schroederi* n. sp. Deutonymph, ventral view. *Scale-bars*: all in micrometres

**Fig. 18:**
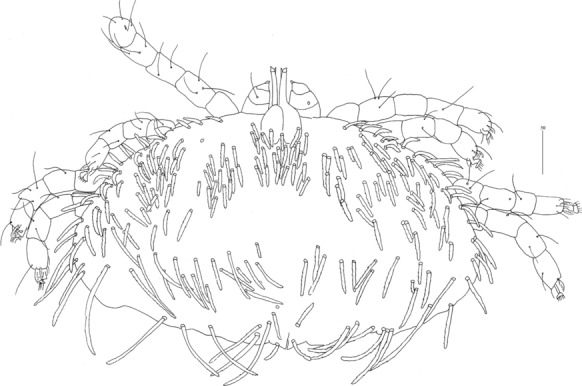
*Neopterygosoma schroederi* n. sp. Protonymph, dorsal view. *Scale-bars*: all in micrometres

**Fig. 19:**
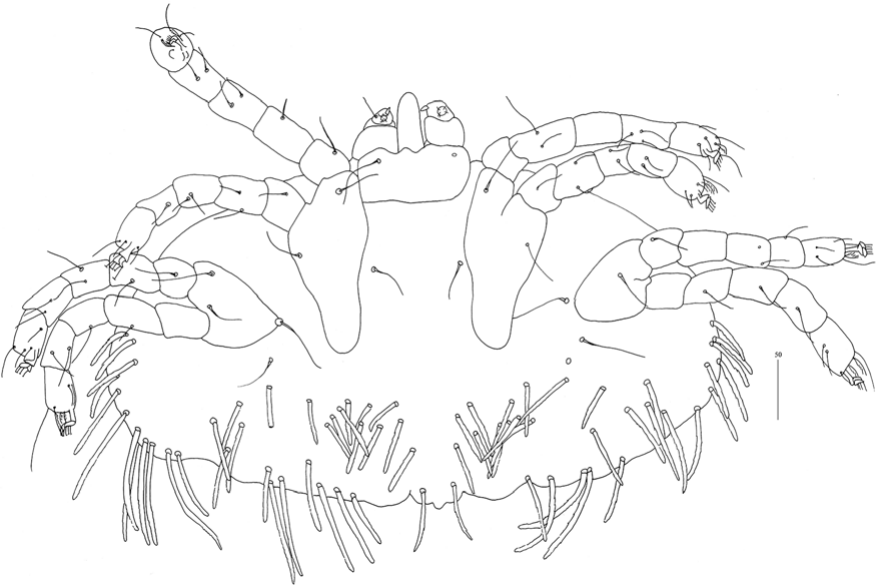
*Neopterygosoma schroederi* n. sp. Protonymph, ventral view. *Scale-bars*: all in micrometres

**Fig. 20:**
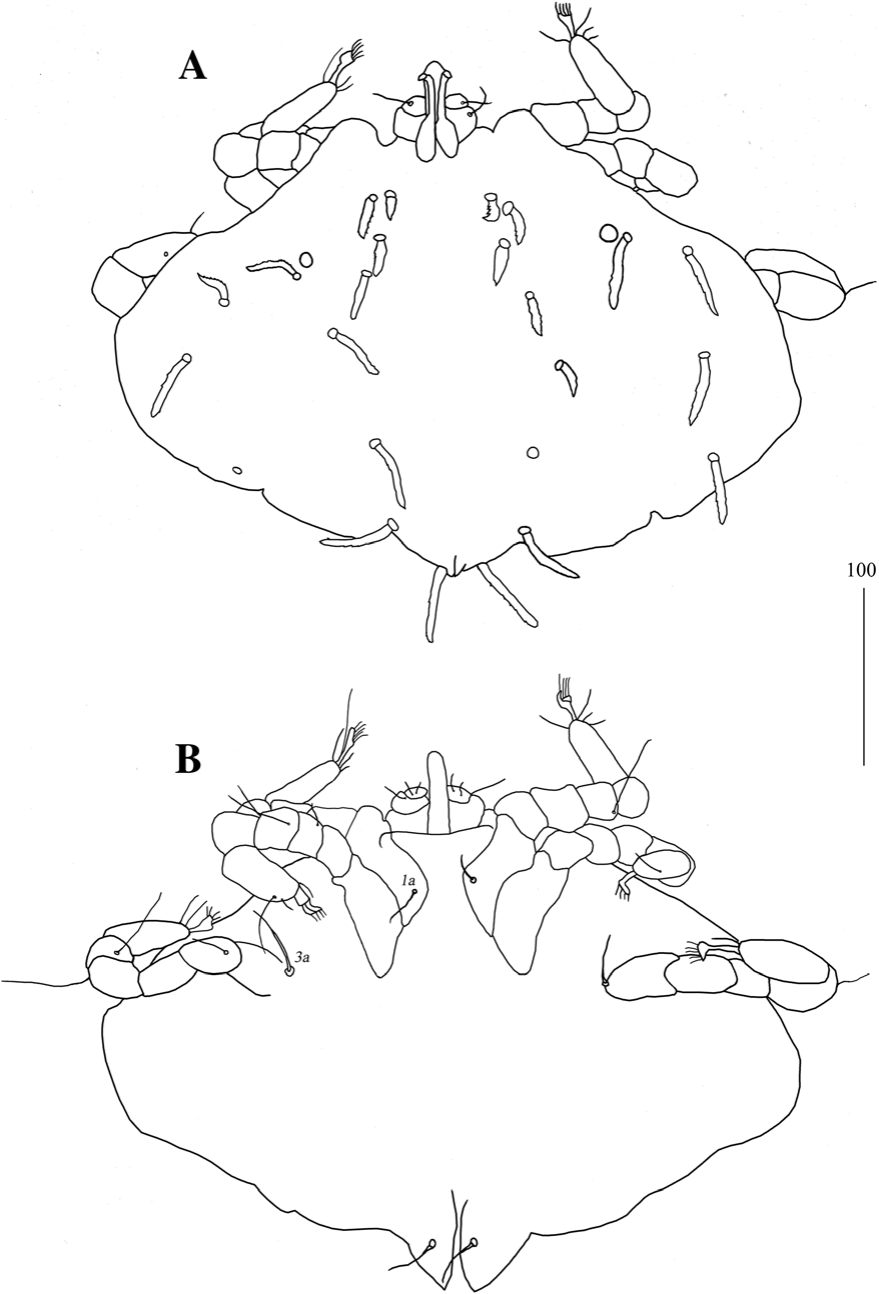
*Neopterygosoma schroederi* n. sp., larva. A, Dorsal view; B, Ventral view. *Scale-bars*: all in micrometres

*Female* [Holotype; Figs. 13–20.] *Gnathosoma*. Chelicerae 195 long. Swollen, proximal part of cheliceral base 100 long; slender distal part 95 long. Fixed cheliceral digit spinous, 10 long. Palpal femur with serrate seta *dF*, 50 long, palpal genu with slightly serrate seta *dG*, 50 long. Palpal tibia *l’Ti* and *l”Ti* with barely discernible serration, seta *vTi* smooth, tarsi with 5 setae and solenidion (Fig. 15A). Hypostome with rounded apex, *c.*145 long. Peritremes with clearly visible chambers, *c.*120 long. Subcapitular seta *n* with barely discernible serration, 125 long. *Idiosoma* 660 long, 985 wide (600–675 long and 980–1,015 wide in 4 paratypes). Dorsum, antero-mid cluster with *c.*60 setae, subequal in length, 15 long; antero-lateral part with *c.*210 pairs of setae on each side, 80–110 long; dorso-median part with *c.*16–22 pairs of setae *dm*, 50–115 long; posterior and postero-lateral parts with 28–35 pairs of peripheral slightly serrate setae, 125–185 long, inserted dorsally and ventrally. Venter, 1 pair of slightly serrate setae *vm*, *c.*60 long, present anterior to genital area; 16 and 21 pairs of serrate setae *vm*, 30–110 long, present laterally to genital area and 15–21 pairs of serrate setae, 35–85 long, present in postero-lateral part of idiosomal venter. Genital series represented by 1 pair of setae *g1* with barely discernible serration, 60 long. Pseudanal series represented by 4 pairs of setae *ps*. Setae *ps1-ps4*, 90, 120, 120 and 130 long, respectively. Setae *ps1* and *ps2* situated ventrally, *ps3* and *ps4* dorsally. *Legs*, coxal setation *1a*, *1b*, *2b*, *3a*, *3b*, *3c* and *3d* arranged in formula 2-1-0-4. Setae *3a* situated outside coxal plates. All coxal setae smooth except for slightly serrate setae *3d*. Setae of trochanters I-IV: 1-1-1-1, femora I-IV: 5-4-3-3, genua I-IV: 5-4-3-3 and tibiae I-IV: 5-5-5-5. Setae *d’FI-IV*, *d”FI-II*, *lFIII*-*IV*, *dGI-IV*, *l’GII-IV* and *dTiI* serrate; *lFI*, *v’FII-IV*, *v”FII*, *l’GI*, *l”GI*, *v’GIII-IV*, *dTiIII-IV*, *l’TiI-IV* and *l”TiI-IV* slightly serrate; *v’GI-II*, *v” GI*, *v’TiI-IV* and *v”TiI-IV* with barely discernible serration.

*Male*. Unknown.

*Deutonymph* [Based on 2 specimens, Figs. 16, 17.] *Gnathosoma* as in female. Chelicerae 155 long; swollen cheliceral part 70 long, slender distal part 85 long. Fixed cheliceral digit spinous, 25 long. Palpal femur with serrate seta *dF* and *dG*, 45–50 and 45–55 long, respectively. Palpal tibia and tarsi as in female. Hypostome 145 long, with small depression present at apex. Peritremes with clearly visible chambers, *c.*90–95 long. Subcapitular seta *n* with barely discernible serration, 65 long. *Idiosoma* 505 long and 770–930 wide. Dorsum, antero-mid cluster with about 50 setae, subequal in length, 20 long; antero-lateral part with *c.*175–195 pairs of setae, 20–75 long, on each side present; dorso-mednian part with 22–25 pairs of setae *dm*, 55–170 long; posterior and postero-lateral parts with 28–35 pairs of peripheral setae, 140–215 long, inserted dorsally and ventrally. Venter with 1 pair of slightly serrate setae *vm*, 60 long, present anteriorly to genital area; 10–18 pairs of serrate setae *vm*, 55–95 long, present lateral to genital area and 15–21 pairs of postero-lateral setae, 35–90 long. Genital series represented by 1 pair of setae *g1* with barely discernible serration, 50–60 long. Pseudanal series represented by 4 pairs of setae *ps*. Setae *ps1-ps4*, 90, 120, 120 and 130 long, respectively. Setae *ps1* and *ps2* situated ventrally, *ps3* and *ps4* situated dorsally. *Legs*, coxal setation *1a*, *1b*, *2b*, *3a*, *3b*, *3c* and *3d* arranged in formula 2-1-0-4. Setae *3a* situated outside coxal plates. All coxal setae smooth except for slightly serrate setae *3d*. Setae of trochanters I-IV (1-1-1-1), femora I-IV (5-4-3-3), genua I-IV (5-4-3-3), tibiae I-IV (5-5-5-5). Setae *d’FI-IV*, *d”FI-II*, *lFIII*-*IV*, *dGI-IV*, *l’GII-IV*, *dTiI* serrate, *lFI*, *v’FII-IV*, *v”FII*, *l’GI*, *l”GI*, *v’GIII-IV*, *dTiIII-IV*, *l’TiI-IV*, *l”TiI-IV* slightly serrate *v’GI-II*, *v” GI*, *v’TiI-IV*, *v”TiI-IV* with barely discernible serration.

*Protonymph* [Based on 5 specimens, Figs. 18, 19.] *Gnathosoma* as in female. Swollen, proximal part of cheliceral base and slender, distal part equal in length, 50 long. Fixed cheliceral digits spinous, 5 long. Palpal femur with serrate seta *dF*, 30 long; palpal genu with filiform setae *dG*, 30 long. Palpal tibia and tarsi as in female. Hypostome *c.*90 long, with small depression present at apex. Peritremes with clearly visible chambers, 65 long. Subcapitular seta *n* 35–45 long. *Idiosoma* 275–365 long, 485–580 wide. Dorsum. Antero-mid-cluster with 26–28 setae, subequal in length, *c.*20 long. Antero-lateral part with *c.*58–79 pairs of setae on each side, 20–45 long; dorso-median part with 8–15 pairs of setae *dm*, 35–50 long; posterior part with 26–35 pairs of peripheral setae, 40–90 long, inserted dorsally and ventrally. Venter with 10–14 pairs of setae *vm*, 30–50 long, present lateral to genital area. Genital setae absent. Pseudanal series represented by 4 pairs of setae *ps*. Setae *ps1* 50–55 long and situated dorsally, setae *ps2* 40–45 long and situated terminally; setae *ps3* and *ps4* 75–95 and 95–105 long, respectively, situated dorsally. *Legs*, coxal setation *1a*, *1b*, *2b*, *3a*, *3b*, *3c* and *3d* arranged in formula 2-1-0-4. Setae of trochanters I-IV: 1-1-1-0, femora I-IV: 5-4-3-2, genua I-IV: 5-5-3-3 and tibiae I-IV: 5-5-5-5. Setae *d’FI-IV*, *d”FII*, *d’GI-IV*, *d”GI-II* and *lGII-III* serrate; *d”FI*, *lFIII*, *lGI* and *lGIV*, slightly serrate; *vTrI-IV*, *v’FI-IV*, *v”FI-II*, *lFI*, *v’GI-IV*, *v”GI-II*, *d’TiI-IV*, *l’TiI-IV*, *l”Ti-IV*, *v’TI-IV* and *v”TiI-IV* with barely discernible serration. Tarsi I-IV as in female.

*Larva* [Based on 1 larva; Fig. 20.] *Gnathosoma* as in female, except for lack of subcapitular setae *n*. *Idiosoma* 240 long, 360 wide. Dorsum with 11 pairs of slightly serrate setae, 10–35 long: 4 pairs situated anteriorly, 4 pairs medio-laterally and 3 pairs posteriorly. Eyes present. Genital slit situated terminally. Genital setae absent. Pseudanal setal series represented by 1 pair of slightly serrate setae *ps1*. *Legs*, coxal setation *1a* and *3a* arranged in formula 1-0-1. All coxal setae filiform. Setae of trochanters I-III: 0-0-0, femora I-III: 2-2-0, genua I-III: 2-3-0 and tibiae I-III: 5-5-5. All setae on each podomere smooth, except for serrate setae *dFI* and *dFII.* Setation of tarsi: I 11 setae (*ft*, *tc’*, *p’*, *it’*, *it”*, *a’*, *a”*, *u’*, *u”*, *vs’*, *vs”*) and solenidion *ω1*; II 8 setae (*tc’*, *p’*, *p”*, *a’*, *a”*, *u’*, *u”*, *vs’*) and *ω1*; III and IV with 8 setae each (*tc’*, *p’*, *p”*, *a’*, *a”*, *u’*, *u”*, *vs’*). Setae *tc’*, *tc”*, *it’* and *it”* of leg I represented by eupathidia, all setae *p’* and *p”* fan-like, setae *a’* and *a”* of legs I-III and setae *u’* and *u”* of leg I smooth; setae *ft*, *tc’*, *vs’*, *vs”*, *u’* and *u”* of legs I-III slightly serrate.

Differential diagnosis

This species is very similar to *Neopterygosoma chilensis* (Fajfer & González-Acuña, 2013) collected from *Liolaemus chilensis* (Lesson) in Chile (Fajfer & González-Acuña, 2013). In females of both species the eyes are present, the fixed cheliceral digit is spinous, and the chaetotaxy of gnathosoma and legs I-IV is the same. However, in *N. schroederi* n. sp. all anterior mid-dorsal setae are subequal in length, setae *3b* are situated outside the coxal plates, and there are four pairs of pseudanal setae *ps* whereas in *N. chilensis* the anterior mid-dorsal setae extend from the anterior to the posterior part of the setal cluster, setae *3b* are situated on the coxal plates, and there are five pairs of pseudanal setae *ps*.

**Species group** ***patagonica***

*Diagnosis*: Body circular, only 1.1–1.2 times wider than long. All legs subequal in length. Postero-medial part of idiosoma with 2 pairs of dorso-median setae *dm.* Peripheral setae few in number and subequal with medial and posterior setae. Leg seta *l’GIV* absent. Setae *tc’* and *tc”* of legs II-IV smooth.

*Microhabitat*: Under the dorsal and lateral scales of the head, belly and tail.

*Distribution and host range*: This group is associated with tree lizard species of the genus *Liolaemus* (Sauria: Liolaemidae) from Argentina.

*Species included*: *Neopterygosoma patagonica* (Dittmar de la Cruz, Morando & Avila, 2004).

***Neopterygosoma patagonica*** **(Dittmar de la Cruz, Morando & Avila, 2004)**

Syn. *Pterygosoma patagonica* Dittmar de la Cruz, Morando & Avila, 2004

*Type-host*: *Liolaemus petrophilus* Donoso-Barros & Cei (Sauria: Liolaemidae).

*Type-locality*: Argentina.

*Type-material* (not examined): The type material is lost (Dittmar de la Cruz, personal communication).

*Neotype* (examined): One female (Reg. no. UKSW-PTE2.1) from *Liolaemus petrophilus* Donoso-Barros & Cei (Sauria: Liolaemidae), Argentina: Chubut Province, Paso de Indios (43°49’41”S, 67°45’21”W; 24.i.2000, coll. M. Morando and L. Avila).

*Other material examined*: Three females from *Liolaemus petrophilus* Donoso-Barros & Cei (Sauria: Liolaemidae), Argentina: Chubut Province, Paso de Indios (43°49’41”S, 67°45’21”W; 24.i.2000, coll. M. Morando and L. Avila); 3 females from *Liolaemus austromendocinus* Cei, Argentina: Mendoza, San Rafael (34°59’29”S, 68°37’24”; 29.iii.2000, coll. M. Morando and L. Avila); 2 females from *Lioleamus rothi* Koslowsky, Argentina: Chubut Province, Telsen (42°22’55.3”S, 67°42’44.8”W; coll. M. Morando and L. Avila). All mites are deposited in the UKSW (UKSW-PTE2.2).

*Records*: Dittmar de la Cruz et al. (2004: p. 2, figures 1–4); Fajfer (Fajfer 2014: p. 2, figures 1–3); Fajfer (2019: p. 422).

Diagnosis

**Fig. 21:**
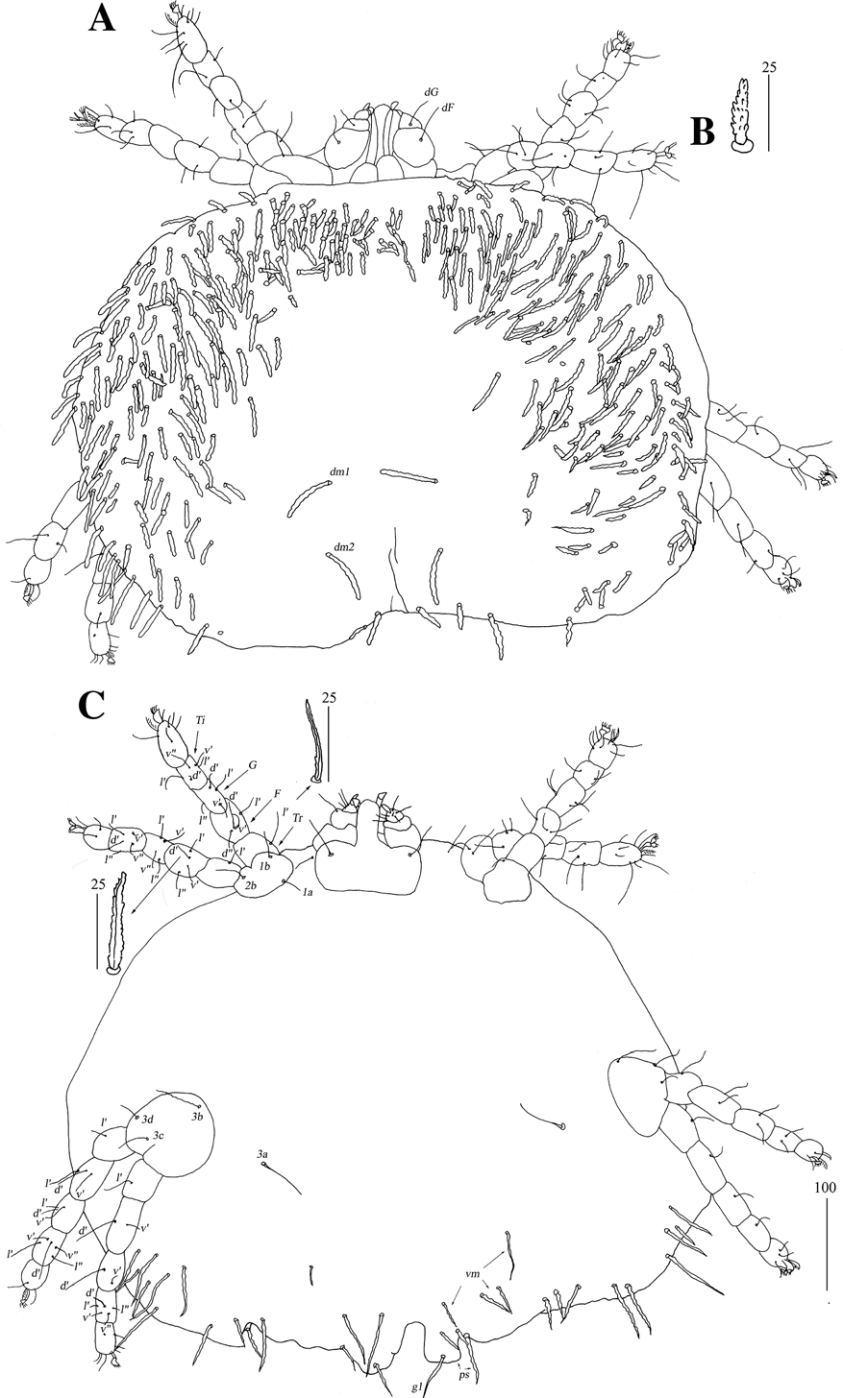
*Neopterygosoma patagonica* (Dittmar de la Cruz, Morando & Avila, 2004), female. A, Dorsal view; B, Ventral view; C, Mid-dorsal seta. *Abbreviations*: *Ti*, tibia, *G*, genu, *F*, femur, *Tr*, trochanter; *d*, dorsal; *l*, lateral; *v*, ventral (after Fajfer, 2014, amended). *Scale-bars*: all in micrometres

**Fig. 22:**
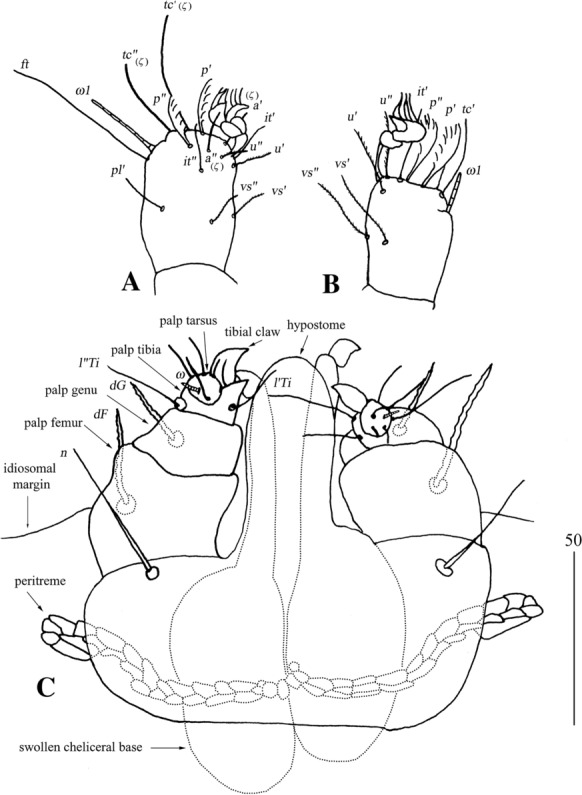
*Neopterygosoma patagonica* (Dittmar de la Cruz, Morando & Avila, 2004), female. A, Tarsus I, lateral view; B, Tarsus II, lateral view; C, Gnathosoma, ventral view (after Fajfer, 2014, amended). *Scale-bars*: all in micrometres

*Female* [Based on the neotype and 8 specimens ex *Liolaemus* sp.; Figs. 21, 22.] *Gnathosoma*. Chelicerae 95–105 long. Swollen, proximal part of cheliceral base slightly shorter than slender, distal part. Fixed cheliceral digit with spinous process. Palpal femur and genu with serrate dorsal seta *dF* and *dG*. Subcapitulum with slightly serrate setae *n*. *Idiosoma* 400–515 long, 500–615 wide. Dorsum with plumose setae. Antero-medial part of idiosoma with 40–50 short setae grouped in cluster. Lateral to this cluster, *c.*170 setae present on each side. Postero-medial part of idiosoma with 2 pairs of dorso-median setae *dm1* and *dm2*. Peripheral series represented by 2 pairs of setae, located near genital region. Venter with 3–6 pairs of slightly serrate setae *vm*. Peripheral part of body with about 9–13 pairs of postero-lateral plumose setae and 4–6 pairs of posterior plumose setae. Genital series represented by 1 pair of slightly serrate setae *g1* and 4 pairs of serrate pseudanal setae *ps*. Chaetotaxy of legs given in Table 1. Coxal setae *3a* and *3d* slightly serrate. All setae on each podomere slender and slightly plumose, except for thick and plumose setae *dFI-IV*. Setation of tarsi I-IV as in Table 2.

### Remarks

In accordance with Article 75.3 of the International Code of Zoological Nomenclature (ICZN 1999, 2012), I designated one of the female specimens as a neotype of *Neopterygosoma patagonica* for the purpose of clarifying the taxonomic status of the species. Originally, this species was insufficiently described by Dittmar de la Cruz et al. (2004). The authors in their type-series included five males (all designated as holotypes) and five females; no data about their exact localities were provided. These specimens were deposited at the Insect Genomics Laboratory, Brigham Young University, Provo, Utah, USA, but to my knowledge, none of the original type-series from this collection are extant (Dittmar de la Cruz, personal communication). Then, a thorough redescription of the species was made by Fajfer (2014); however, no neotype was designated by the author. Therefore, based on the close morphological similarity of the newly collected mites and the specimens redescribed by Fajfer (2014) to the original description of the species (Dittmar de la Cruz et al., 2004), I designate a neotype (Reg. no. UKSW-PTE2.1) and present a diagnosis of the species above.

**Key to species of** ***Neopterygosoma*** **Fajfer, 2019**

1. Body much wider than long (1.5–1.8 times). Second pair of legs discernibly shorter than others. Peripheral setae much longer than dorsal setae situated medially and laterally. Leg setae *l’GIV* present ………………………………………………. 2 (*chilensis* group)
2. Body only slightly wider than long (1.1–1.3 times). Legs I-IV subegual in length. Peripheral setae subequal with medial and lateral setae on idiosomal dorsum. Leg setae *l’GIV* absent ……………………………………………………… *N. patagonica* (*patagonica* group)
3. Five setae on genu I and 3 on femur IV ………………………………………………………………. 3
4. Four setae on genu I and 2 setae on femur IV ………………………………………. *N.* *formosus*
5. Four setae on femur II ………………………………………………………………………………………. 4
6. Five setae on femur II ……………………………………………………………………………………….. 5
7. Pseudanal setae 5 ………………………………………………………………………………. *N. chilensis*
8. Pseudanal setae 4 …………………………………………………………………… *N. schroederi* n. sp.
9. Fixed cheliceral digit spinous ……………………………………………………….. *N. cyanogasteri*
10. Fixed cheliceral digit reduced to rounded structure ………………………………………………. 6
11. Coxal fields I with 2 setae. Gnathosoma situated apically ……………………………………… 7
12. Coxal fields I with 3 setae. Gnathosoma displaced on dorsal side ………………… *N. ovata*
13. Antero-median setae increase in length from the anterior to the posterior part of setal cluster. Setae *a’* and *a”* of tarsi I slightly serrate. Setae *v’TrI-IV* serrate. Setae *3a* smooth …………………………………………………………………………………………….. *N. levissima*
14. Antero-median setae subequal in length. Setae *a’* and *a”* of tarsi I smooth. Setae *v’TrI-IV* with barely discernible serration. Setae *3a* serrate …………………………………. *N. ligare*
